# 2,8-Dimethyl­tricyclo­[5.3.1.1^3,9^]dodecane-*syn*-2,*syn*-8-diol–propanoic acid (1/1)

**DOI:** 10.1107/S1600536809016547

**Published:** 2009-05-14

**Authors:** Yuji Mizobe, Roger Bishop, Donald C. Craig, Marcia L. Scudder

**Affiliations:** a School of Chemistry, University of New South Wales, Sydney, New South Wales 2052, Australia

## Abstract

The racemic title compound, C_14_H_24_O_2_·C_3_H_6_O_2_, crystallizes in the monoclinic space group *P*2_1_/*c* as a 1:1 diol/carboxylic acid cocrystal, *A*–*B*. The lattice incorporates infinite chains of the alcohol–carboxylic acid–alcohol supra­molecular synthon, (⋯O—H⋯O=C(*R*)—O—H⋯O—H⋯), in which the hydrogen-bonded mol­ecules (*A*—*B*—*A*)_*n*_ surround a pseudo-threefold screw axis. The carboxylic acid group functions like an extended alcohol hydr­oxy group. Each diol, *A*, takes part in two such threefold screw arrangements, leading to a hydrogen-bonded layer structure, with adjacent layers containing diol mol­ecules of opposite handedness. The central C atom of the propano bridge is disordered over two sites of occupancies 0.75 (1) and 0.25 (1). The methyl group of the propanoic acid molecule is disordered over two sites of occupancies 0.68 (1) and 0.32 (1).

## Related literature

For related literature on the diol component of the title compound, see: Bishop (2009 ▶); Dance *et al.* (1986 ▶). Two members of this diol family have been found previously to form such 1:1 compounds with carboxylic acids, see: Alshahateet *et al.* (2004 ▶); Yue *et al.* (2006 ▶).
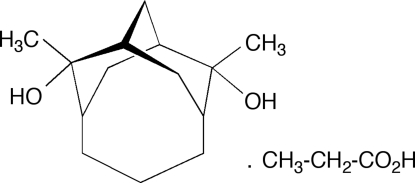


## Experimental

### 

#### Crystal data

C_14_H_24_O_2_·C_3_H_6_O_2_
*M* *_r_* = 298.4
Monoclinic, 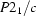

*a* = 7.390 (4) Å
*b* = 13.218 (5) Å
*c* = 18.469 (8) Å
β = 110.23 (2)°
*V* = 1693 (1) Å^3^
*Z* = 4
Mo *K*α radiation
μ = 0.08 mm^−1^
*T* = 294 K
0.10 mm (radius)

#### Data collection

Enraf–Nonius CAD-4 diffractometer
Absorption correction: none
3188 measured reflections
2942 independent reflections
1786 reflections with *I* > 2σ(*I*)
*R*_int_ = 0.014
1 standard reflections frequency: 30 min intensity decay: 29%

#### Refinement

*R*[*F* ^2^ > 2σ(*F* ^2^)] = 0.056
*wR*(*F* ^2^) = 0.070
*S* = 1.32
2942 reflections
199 parameters
H-atom parameters constrained
Δρ_max_ = 0.39 e Å^−3^
Δρ_min_ = −0.41 e Å^−3^

### 

Data collection: *CAD-4 Software* (Enraf–Nonius, 1989 ▶); cell refinement: *CAD-4 Software*; data reduction: local program; program(s) used to solve structure: *SIR92* (Altomare *et al.*, 1994 ▶); program(s) used to refine structure: *RAELS* (Rae, 2000 ▶); molecular graphics: *ORTEP-3* (Farrugia, 1997 ▶) and *CrystalMaker* (Palmer, 2005 ▶); software used to prepare material for publication: local programs.

## Supplementary Material

Crystal structure: contains datablocks global, I. DOI: 10.1107/S1600536809016547/hg2504sup1.cif

Structure factors: contains datablocks I. DOI: 10.1107/S1600536809016547/hg2504Isup2.hkl

Additional supplementary materials:  crystallographic information; 3D view; checkCIF report

## Figures and Tables

**Table 1:** Hydrogen-bond geometry (Å, °)

*D*—H⋯*A*	*D*—H	H⋯*A*	*D*⋯*A*	*D*—H⋯*A*
O1—H101⋯O2*P*^i^	1.00	1.82	2.822 (3)	180
O2—H102⋯O1^ii^	1.00	1.75	2.746 (3)	180
O1*P*—H101*P*⋯O2	1.00	1.64	2.635 (3)	180

Symmetry codes: (i) 
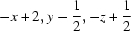
; (ii) 
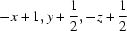
.

**Fig. 1.**
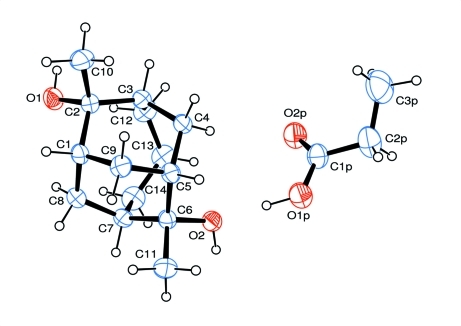
Molecular structure of the A and B components of the title compound, with ellipsoids drawn at the 30% probability level.

**Fig. 2.**
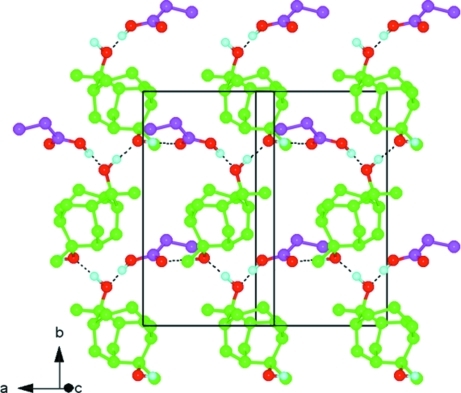
One layer of the structure showing the intermolecular hydrogen bonding linking A and B molecules in chains. C atoms of the propanoic acid are coloured pink.

**Fig. 3.**
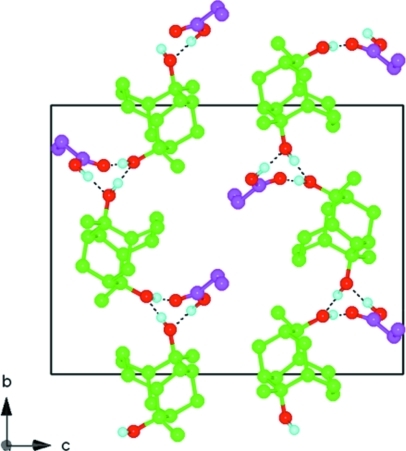
The orthogonal view showing the pseudo 31 symmetric nature of the arrangement in two adjacent layers. C atoms of the propanoic acid are coloured pink.

## supplementary crystallographic information

### Comment

The diol component, *A*, of the title compound, *A—B*, is a member of the helical tubuland host family, a major characteristic of which is formation of lattice inclusion compounds in the chiral space group *P*3_1_21 (or its enantiomorph *P*3_2_21) (Bishop, 2009). *A* forms this structure when crystallized from non-protic solvents (Dance *et al.*, 1986). Some, but by no means all, of this family of diols can also form hydrogen-bonded co-crystals when crystallized from protic solvents. Two members of this diol family have been found previously to form such 1:1 compounds with carboxylic acids (Alshahateet *et al.*, 2004; Yue *et al.*, 2006). These co-crystals utilize infinite chains of an alcohol–carboxylic acid–alcohol supramolecular sython, (···O—H···O═ C(*R*)—O—H···O—H···), in which the carboxylic acid group behaves as if it were an extended alcohol hydroxy group. The diol, *A*, in the title compound is now found to be the third helical tubuland diol to behave in this manner (Fig. 1). Its 1:1 co-crystals with propanoic acid, *A—B*, contain chains of hydrogen-bonded molecules (*A—B—A-*)*_n_* surrounding pseudo-threefold screw axes resulting in formation of chiral layers as each diol, *A*, hydrogen bonds within two such threefold screw arrangements (Figs. 2 and 3). Adjacent layers contain diol molecules with the opposite handedness. The resultant lattice is essentially isostructural with the previous examples in *P*2_1_/*c* found to use this novel supramolecular synthon.

### Experimental

Racemic 2,8-dimethyltricyclo[5.3.1.1^3,9^]dodecane-*syn*-2,*syn*-8-diol was prepared as described (Dance *et al.*, 1986) and the X-ray quality co-crystals obtained by slow concentration of a propanoic acid solution.

### Refinement

The central C atom of the propano bridge (C13) was disordered over two sites of occupancies 0.75 (1) and 0.25. For the propanoic acid molecules, the methyl group, C3P, was disordered over two sites of occupancies 0.68 (1) and 0.32. H atoms attached to C were included at calculated positions (C—H = 1.0 Å). The disorder of C13 was taken into account when calculating the H atom positions and occupancies for C13 and the adjacent C12 and C14. The hydroxy H atoms were located on a difference map, and were then fixed at a position along the O···O vector with O—H = 1.0 Å. All H atoms were refined with isotropic thermal parameters equivalent to those of the atom to which they were bonded.

### Crystal data

**Table:**

C_14_H_24_O_2_·C_3_H_6_O_2_	*F*(000) = 656.0
*M**_r_* = 298.4	*D*_x_ = 1.17 Mg m^−^^3^
Monoclinic, *P*2_1_/*c*	Mo *K*α radiation, λ = 0.71073 Å
*a* = 7.390 (4) Å	Cell parameters from 11 reflections
*b* = 13.218 (5) Å	θ = 11–12°
*c* = 18.469 (8) Å	µ = 0.08 mm^−^^1^
β = 110.23 (2)°	*T* = 294 K
*V* = 1693 (1) Å^3^	Irregular, colourless
*Z* = 4	0.10 mm (radius)

### Data collection

**Table:**

Enraf–Nonius CAD-4 diffractometer	θ_max_ = 25°
ω/2θ scans	*h* = 0→8
3188 measured reflections	*k* = 0→15
2942 independent reflections	*l* = −22→22
1786 reflections with *I* > 2σ(*I*)	1 standard reflections every 30 min
*R*_int_ = 0.014	intensity decay: 29%

### Refinement

**Table:**

Refinement on *F*	0 restraints
*R*[*F*^2^ > 2σ(*F*^2^)] = 0.056	H-atom parameters constrained
*wR*(*F*^2^) = 0.070	*w* = 1/[σ^2^(*F*) + 0.0004*F*^2^]
*S* = 1.32	(Δ/σ)_max_ = 0.003
2942 reflections	Δρ_max_ = 0.39 e Å^−^^3^
199 parameters	Δρ_min_ = −0.41 e Å^−^^3^

### Fractional atomic coordinates and isotropic or equivalent isotropic displacement parameters (Å^2^)

**Table:**

	*x*	*y*	*z*	*U*_iso_*/*U*_eq_	Occ. (<1)
O1	0.7617 (2)	0.2866 (1)	0.2685 (1)	0.0659 (5)	
O2	0.4331 (2)	0.66382 (11)	0.16336 (9)	0.0559 (5)	
C1	0.4896 (3)	0.3428 (2)	0.1636 (1)	0.0495 (6)	
C2	0.7107 (3)	0.3385 (2)	0.1951 (1)	0.0496 (6)	
C3	0.8088 (3)	0.4441 (2)	0.2035 (1)	0.0513 (6)	
C4	0.6996 (3)	0.5155 (2)	0.1356 (1)	0.0503 (6)	
C5	0.4789 (3)	0.5105 (2)	0.1032 (1)	0.0475 (6)	
C6	0.3662 (3)	0.5599 (2)	0.1493 (1)	0.0472 (6)	
C7	0.3899 (3)	0.5039 (2)	0.2262 (1)	0.0528 (6)	
C8	0.3898 (4)	0.3878 (2)	0.2161 (1)	0.0579 (7)	
C9	0.4195 (3)	0.4001 (2)	0.0871 (1)	0.0536 (6)	
C10	0.7824 (4)	0.2728 (2)	0.1425 (2)	0.0718 (8)	
C11	0.1519 (4)	0.5658 (2)	0.1009 (2)	0.0656 (8)	
C12	0.8701 (4)	0.4943 (2)	0.2831 (2)	0.0695 (8)	
C13	0.7455 (5)	0.5768 (3)	0.2979 (2)	0.067 (1)	0.75
C13'	0.7388 (9)	0.4872 (7)	0.3304 (4)	0.067 (1)	0.25
C14	0.5481 (5)	0.5443 (2)	0.2994 (1)	0.0707 (8)	
O1P	0.5377 (3)	0.7775 (1)	0.0674 (1)	0.0740 (6)	
O2P	0.8391 (3)	0.7734 (2)	0.1482 (1)	0.0801 (6)	
C1P	0.7228 (4)	0.7995 (2)	0.0878 (2)	0.0667 (7)	
C2P	0.7710 (5)	0.8619 (3)	0.0287 (2)	0.096 (1)	
C3P	0.9587 (9)	0.8359 (5)	0.0180 (3)	0.119 (2)	0.68
C3'P	0.6746 (18)	0.9630 (8)	0.0220 (6)	0.119 (2)	0.32
H101	0.9031	0.2819	0.2980	0.066	
H102	0.3622	0.7085	0.1882	0.056	
HC1	0.4427	0.2716	0.1523	0.049	
HC3	0.9335	0.4301	0.1955	0.051	
H1C4	0.7466	0.5001	0.0921	0.050	
H2C4	0.7357	0.5865	0.1536	0.050	
HC5	0.4377	0.5454	0.0520	0.047	
HC7	0.2666	0.5181	0.2354	0.053	
H1C8	0.2520	0.3657	0.1953	0.058	
H2C8	0.4535	0.3580	0.2686	0.058	
H1C9	0.2759	0.3951	0.0636	0.054	
H2C9	0.4795	0.3706	0.0509	0.054	
H1C10	0.7499	0.3061	0.0909	0.072	
H2C10	0.9254	0.2643	0.1660	0.072	
H3C10	0.7188	0.2050	0.1360	0.072	
H1C11	0.0979	0.4958	0.0894	0.066	
H2C11	0.0820	0.6035	0.1301	0.066	
H3C11	0.1354	0.6018	0.0514	0.066	
H1C12	1.0007	0.5242	0.2929	0.069	0.75
H2C12	0.8794	0.4394	0.3215	0.069	0.75
H1'C12	0.8886	0.5679	0.2752	0.069	0.25
H2'C12	0.9963	0.4635	0.3147	0.069	0.25
H1C13	0.7237	0.6289	0.2565	0.067	0.75
H2C13	0.8185	0.6079	0.3491	0.067	0.75
H1C13'	0.8114	0.5142	0.3831	0.067	0.25
H2C13'	0.7085	0.4141	0.3342	0.067	0.25
H1C14	0.5727	0.4897	0.3391	0.071	0.75
H2C14	0.4921	0.6047	0.3166	0.071	0.75
H1'C14	0.4920	0.5456	0.3415	0.071	0.25
H2'C14	0.5785	0.6150	0.2880	0.071	0.25
H101P	0.4980	0.7344	0.1038	0.074	
H1C2P	0.7770	0.9345	0.0447	0.096	0.68
H2C2P	0.6649	0.8527	−0.0221	0.096	0.68
H1'C2P	0.7236	0.8267	−0.0224	0.096	0.32
H2'C2P	0.9138	0.8713	0.0452	0.096	0.32
H1C3P	0.9777	0.8811	−0.0222	0.119	0.68
H2C3P	1.0675	0.8456	0.0679	0.119	0.68
H3C3P	0.9554	0.7638	0.0011	0.119	0.68
H1C3P'	0.7056	1.0050	−0.0172	0.119	0.32
H2C3P'	0.5318	0.9533	0.0056	0.119	0.32
H3C3P'	0.7219	0.9979	0.0732	0.119	0.32

### Atomic displacement parameters (Å^2^)

**Table:**

	*U*^11^	*U*^22^	*U*^33^	*U*^12^	*U*^13^	*U*^23^
O1	0.052 (1)	0.071 (1)	0.071 (1)	−0.0012 (9)	0.0151 (9)	0.0241 (9)
O2	0.060 (1)	0.046 (1)	0.068 (1)	−0.0018 (8)	0.0298 (8)	−0.0045 (8)
C1	0.049 (1)	0.041 (1)	0.059 (1)	−0.008 (1)	0.021 (1)	−0.002 (1)
C2	0.050 (1)	0.045 (1)	0.057 (1)	−0.001 (1)	0.022 (1)	0.003 (1)
C3	0.046 (1)	0.048 (1)	0.061 (2)	−0.002 (1)	0.020 (1)	0.000 (1)
C4	0.051 (1)	0.047 (1)	0.060 (2)	−0.002 (1)	0.028 (1)	0.000 (1)
C5	0.051 (1)	0.049 (1)	0.044 (1)	−0.003 (1)	0.019 (1)	0.001 (1)
C6	0.047 (1)	0.043 (1)	0.054 (1)	−0.003 (1)	0.020 (1)	−0.002 (1)
C7	0.053 (2)	0.058 (1)	0.056 (1)	0.003 (1)	0.030 (1)	0.005 (1)
C8	0.054 (2)	0.057 (2)	0.070 (2)	−0.002 (1)	0.031 (1)	0.007 (1)
C9	0.054 (2)	0.052 (1)	0.053 (2)	−0.003 (1)	0.016 (1)	−0.005 (1)
C10	0.067 (2)	0.058 (2)	0.099 (2)	0.005 (1)	0.039 (2)	−0.010 (2)
C11	0.047 (2)	0.070 (2)	0.074 (2)	0.002 (1)	0.014 (1)	0.004 (1)
C12	0.059 (2)	0.067 (2)	0.068 (2)	0.000 (1)	0.003 (1)	−0.008 (1)
C13	0.069 (2)	0.068 (2)	0.055 (2)	−0.005 (2)	0.008 (2)	−0.015 (2)
C13'	0.069 (2)	0.068 (2)	0.055 (2)	−0.005 (2)	0.008 (2)	−0.015 (2)
C14	0.087 (2)	0.077 (2)	0.049 (2)	0.002 (2)	0.025 (1)	−0.008 (1)
O1P	0.068 (1)	0.083 (1)	0.066 (1)	−0.010 (1)	0.0154 (9)	0.008 (1)
O2P	0.067 (1)	0.087 (1)	0.074 (1)	−0.013 (1)	0.008 (1)	0.011 (1)
C1P	0.073 (2)	0.058 (2)	0.067 (2)	−0.012 (2)	0.022 (2)	−0.002 (1)
C2P	0.105 (3)	0.099 (3)	0.084 (2)	−0.020 (2)	0.031 (2)	0.017 (2)
C3P	0.136 (5)	0.143 (5)	0.083 (3)	−0.002 (4)	0.046 (3)	0.024 (3)
C3'P	0.136 (5)	0.143 (5)	0.083 (3)	−0.002 (4)	0.046 (3)	0.024 (3)

### Geometric parameters (Å, °)

**Table:**

O1—C2	1.448 (3)	C11—H2C11	1.000
O1—H101	1.000	C11—H3C11	1.000
O2—C6	1.453 (3)	C12—C13	1.512 (4)
O2—H102	1.000	C12—C13'	1.516 (5)
C1—C2	1.535 (3)	C12—H1C12	1.000
C1—C8	1.527 (3)	C12—H2C12	1.000
C1—C9	1.527 (3)	C12—H1'C12	1.000
C1—HC1	1.000	C12—H2'C12	1.000
C2—C3	1.555 (3)	C13—C14	1.531 (4)
C2—C10	1.528 (3)	C13—H1C13	1.000
C3—C4	1.555 (3)	C13—H2C13	1.000
C3—C12	1.532 (3)	C13'—C14	1.525 (5)
C3—HC3	1.000	C13'—H1C13'	1.000
C4—C5	1.532 (3)	C13'—H2C13'	1.000
C4—H1C4	1.000	C14—H1C14	1.000
C4—H2C4	1.000	C14—H2C14	1.000
C5—C6	1.529 (3)	O1P—C1P	1.319 (3)
C5—C9	1.524 (3)	O1P—H101P	1.000
C5—HC5	1.000	O2P—C1P	1.199 (3)
C6—C7	1.556 (3)	C1P—C2P	1.506 (4)
C6—C11	1.528 (3)	C2P—C3P	1.507 (6)
C7—C8	1.547 (3)	C2P—C3'P	1.499 (8)
C7—C14	1.545 (4)	C2P—H1C2P	1.000
C7—HC7	1.000	C2P—H2C2P	1.000
C8—H1C8	1.000	C2P—H1'C2P	1.000
C8—H2C8	1.000	C2P—H2'C2P	1.000
C9—H1C9	1.000	C3P—H1C3P	1.000
C9—H2C9	1.000	C3P—H2C3P	1.000
C10—H1C10	1.000	C3P—H3C3P	1.000
C10—H2C10	1.000	C3'P—H1C3P'	1.000
C10—H3C10	1.000	C3'P—H2C3P'	1.000
C11—H1C11	1.000	C3'P—H3C3P'	1.000
			
C2—O1—H101	115.1	C6—C11—H1C11	109.5
C6—O2—H102	116.1	C6—C11—H2C11	109.5
C2—C1—C8	117.3 (2)	C6—C11—H3C11	109.5
C2—C1—C9	110.2 (2)	H1C11—C11—H2C11	109.5
C2—C1—HC1	106.9	H1C11—C11—H3C11	109.5
C8—C1—C9	108.1 (2)	H2C11—C11—H3C11	109.5
C8—C1—HC1	106.9	C3—C12—C13	119.4 (2)
C9—C1—HC1	106.9	C3—C12—C13'	119.4 (4)
O1—C2—C1	105.7 (2)	C3—C12—H1C12	106.9
O1—C2—C3	111.7 (2)	C3—C12—H2C12	106.9
O1—C2—C10	106.9 (2)	C3—C12—H1'C12	106.9
C1—C2—C3	113.8 (2)	C3—C12—H2'C12	106.9
C1—C2—C10	109.8 (2)	C13—C12—H1C12	106.9
C3—C2—C10	108.8 (2)	C13—C12—H2C12	106.9
C2—C3—C4	111.7 (2)	C13'—C12—H1'C12	106.9
C2—C3—C12	117.2 (2)	C13'—C12—H2'C12	106.9
C2—C3—HC3	103.9	H1C12—C12—H2C12	109.5
C4—C3—C12	114.2 (2)	H1'C12—C12—H2'C12	109.5
C4—C3—HC3	103.9	C12—C13—C14	116.4 (3)
C12—C3—HC3	103.9	C12—C13—H1C13	107.7
C3—C4—C5	118.3 (2)	C12—C13—H2C13	107.7
C3—C4—H1C4	107.2	C14—C13—H1C13	107.7
C3—C4—H2C4	107.2	C14—C13—H2C13	107.7
C5—C4—H1C4	107.2	H1C13—C13—H2C13	109.5
C5—C4—H2C4	107.2	C12—C13'—C14	116.5 (4)
H1C4—C4—H2C4	109.5	C12—C13'—H1C13'	107.7
C4—C5—C6	118.4 (2)	C12—C13'—H2C13'	107.7
C4—C5—C9	108.3 (2)	C14—C13'—H1C13'	107.7
C4—C5—HC5	106.5	C14—C13'—H2C13'	107.7
C6—C5—C9	110.0 (2)	H1C13'—C13'—H2C13'	109.5
C6—C5—HC5	106.5	C7—C14—C13	121.3 (2)
C9—C5—HC5	106.5	C7—C14—C13'	118.6 (4)
O2—C6—C5	106.6 (2)	C7—C14—H1C14	106.4
O2—C6—C7	111.3 (2)	C7—C14—H2C14	106.4
O2—C6—C11	106.0 (2)	C13—C14—H1C14	106.4
C5—C6—C7	113.3 (2)	C13—C14—H2C14	106.4
C5—C6—C11	110.5 (2)	H1C14—C14—H2C14	109.5
C7—C6—C11	109.0 (2)	C1P—O1P—H101P	116.7
C6—C7—C8	111.5 (2)	O1P—C1P—O2P	122.8 (3)
C6—C7—C14	116.5 (2)	O1P—C1P—C2P	113.2 (3)
C6—C7—HC7	104.2	O2P—C1P—C2P	124.0 (3)
C8—C7—C14	114.6 (2)	C1P—C2P—C3P	115.4 (3)
C8—C7—HC7	104.2	C1P—C2P—C3'P	108.8 (5)
C14—C7—HC7	104.2	C1P—C2P—H1C2P	108.0
C1—C8—C7	118.9 (2)	C1P—C2P—H2C2P	108.0
C1—C8—H1C8	107.1	C1P—C2P—H1'C2P	109.6
C1—C8—H2C8	107.1	C1P—C2P—H2'C2P	109.6
C7—C8—H1C8	107.1	H1C2P—C2P—H2C2P	109.5
C7—C8—H2C8	107.1	H1'C2P—C2P—H2'C2P	109.5
H1C8—C8—H2C8	109.5	C2P—C3P—H1C3P	109.5
C1—C9—C5	108.2 (2)	C2P—C3P—H2C3P	109.5
C1—C9—H1C9	109.8	C2P—C3P—H3C3P	109.5
C1—C9—H2C9	109.8	H1C3P—C3P—H2C3P	109.5
C5—C9—H1C9	109.8	H1C3P—C3P—H3C3P	109.5
C5—C9—H2C9	109.8	H2C3P—C3P—H3C3P	109.5
H1C9—C9—H2C9	109.5	C2P—C3'P—H1C3P'	109.5
C2—C10—H1C10	109.5	C2P—C3'P—H2C3P'	109.5
C2—C10—H2C10	109.5	C2P—C3'P—H3C3P'	109.5
C2—C10—H3C10	109.5	H1C3P'—C3'P—H2C3P'	109.5
H1C10—C10—H2C10	109.5	H1C3P'—C3'P—H3C3P'	109.5
H1C10—C10—H3C10	109.5	H2C3P'—C3'P—H3C3P'	109.5
H2C10—C10—H3C10	109.5		

### Hydrogen-bond geometry (Å, °)

**Table:**

*D*—H···*A*	*D*—H	H···*A*	*D*···*A*	*D*—H···*A*
O1—H101···O2P^i^	1.00	1.82	2.822 (3)	180
O2—H102···O1^ii^	1.00	1.75	2.746 (3)	180
O1P—H101P···O2	1.00	1.64	2.635 (3)	180

Symmetry codes: (i) −*x*+2, *y*−1/2, −*z*+1/2; (ii) −*x*+1, *y*+1/2, −*z*+1/2.
